# The concept that went viral: Using machine learning to discover charisma in the wild

**DOI:** 10.1111/1468-4446.13146

**Published:** 2024-09-14

**Authors:** Paul Joosse, Yulin Lu

**Affiliations:** ^1^ Department of Sociology University of Hong Kong Hong Kong Hong Kong; ^2^ Lund University Lund Sweden

**Keywords:** charisma, machine learning, Max Weber, power, rizz

## Abstract

The term “charisma” is recognized as sociology's most successful export to common speech. While sociologists habitually dismiss popular uses of the word, we address its vernacularity head on as a worthy object of study and as a potential resource for conceptual development. Using machine learning, we locate “charisma” within the wider discursive field out of which it arises (and continues to arise) across four corpora; namely: Weber’s major writings; social scientific research (123,531 JSTOR articles); and social media (“X”) posts containing of “charisma” (*n*=77,161) and its 2023 variant, “rizz” (*n*=85,869). By capturing meaning structures that discursively suspend “charisma” across multiple dimensions, we discern three spectra that help to distinguish charisma’s sociological and non‐sociological uses. Spectrum one differentiates perspectives which see charisma as having either a structural or individual‐level range of efficacy. Spectrum two differentiates indifferent/analytical perspectives on charisma from perspectives which see it as desirable but also morally conservative. Spectrum three differentiates between relational and individualized ontologies for charisma. We find that, rather than hewing closely to the Weberian formulation, social scientific uses exist in an intermediate position vis‐à‐vis these three spectra. Thus, scholars participate in what they otherwise criticize as charisma’s vulgarization. The article concludes with recommendations for how to constructively interact with ‘popular charisma.’

## 1 INTRODUCTION

Whatever sense of ownership sociologists may feel toward “charisma,” it is above all a creature of vernacular speech. In the ancient Greek (*χάρισμα*), it was a descriptor for signs of the Holy Spirit's presence within the burgeoning Jesus movement (1 Corinthians 12:4–19; Romans 12:1–8; also Acts 1:4–5; 2:1–13). Speaking in tongues, giving prophesy, faith healing—these *charismata* were centerpieces for a public spectacle that traveled, first as vectors of fascination through crowds, and later as waves of enthusiasm through the Greco‐Roman world. Even our surviving written sources for the term, which today are viewed somewhat stuffily as “scripture,” were in the first instance popular missives that would have been read aloud to gathered masses.^1^ Only later did “charisma” fall into obscurity, persisting in the academic guise of arcane theology (Sohm, 1895(1909); see Joosse, 2014; Smith, 1998).^2^

When Max Weber repurposed the term, featuring it prominently in his later work,^3^ it nevertheless initially remained obscure. To be sure, his reconstruction of charisma prompted fascination and debate among intellectuals in interwar Germany (Zelinsky, 2023). But it would be nearly two decades after Weber's death, amid the explanatory demands created by the “Hitler movement” (Abel, 1937; Becker, 1946; Gerth, 1940) that the term would ascend to become the buzzword we know today.

Thus, while sociologists are fond of claiming credit on behalf of their founder for this contribution to popular speech—to say that “Weber gave us charisma”^4^—Joshua Derman (2012) has artfully argued for something approaching the converse proposition: that actually, *charisma gave us Weber*; that Weber owes *his* prominence in the history of ideas, and especially his canonization in English‐speaking sociology, to the fact that at various times and in a variety of ways, the public put “charisma” to service for thinking through its disparate set of concerns.^5^

**FIGURE 1:**
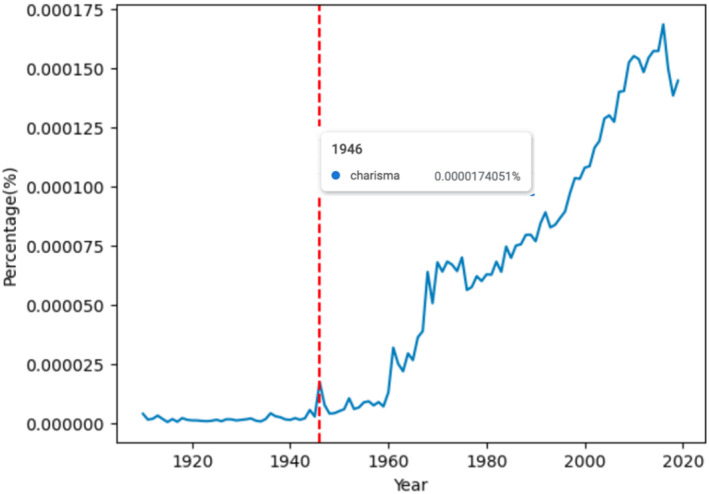
Frequency of “charisma” in Google Books Ngram (English), 1905–2019. The red line indicates the publication of *From Max* Weber (1946), the book through which much of the English‐speaking world was first introduced to Weber's writings. *Source*: Google Books Ngram Viewer, https://books.google.com/ngrams/info.

Today, while the notion of “disciplinary fathers” has lost much of its charm among those who would prefer to decenter canonical figures (e.g., Connell, 2007; Lengermann & Niebrugge, 2006; Morris, 2015), “charisma” as a term has never been more popular (see Figure 1). As Joseph Zadeh (2023) recently observed:

its usage in English [is] still on a steep upward trend. And not just in English: Charisma has migrated to Chinese in its Western pronunciation, to Japanese as ‘karisuma’ and to Spanish, French and Italian as ‘carisma,’ ‘charisme’ and ‘carisma’ respectively. The wholesale migration of the word in exact or close to its original form suggests that no equivalent previously existed in those languages to express its magnetic and mysterious quality.

On Tiktok, an abbreviation of the term—“rizz”—recently went viral, attracting 36 billion views as of this writing. Denoting the ability attract a love interest, “rizz” became such a phenomenon that Oxford dictionary named it “Word of the Year” for 2023 (Oxford, 2023).

One can see by this record that there is something of a “people's history” figuring heavily into the etiology of charisma. One might further wonder how this history is viewed when academics tell the story, and whether academics decry the loose usage of a term, that in scholarly hands, serves as a precision instrument. A few examples will suffice to show that, in charisma's case, the alleged violations are diverse and flagrant. Charisma has been “watered down,” often to nothing more than “trendy rhetoric” (Collins, 2020, p. 1; see also Bendix, 1967). “In ordinary language,” charisma retains only “a weak meaning…synonymous with…the mere communication process” (Abbruzzese, 2001, p. 1657). “In becoming such a popular term” charisma has lost the “revolutionary sense that Weber had so strenuously emphasized” (Adair‐Toteff, 2021, p. 7). “A common misunderstanding, …especially in popular discourse,” is the “inherency fallacy”—the essentialist tendency to view charisma as a personal trait (Joosse, 2014, p. 272; see also Bourdieu, 1987). Even learned commenters from outside of the academy have at times felt compelled to mount a defense. In 1959, writers in *The Times Literary Supplement* opined that “Max Weber would doubtless have been horrified, had he lived, to see the uses to which his doctrine of the “charisma” which descends upon the born leader was to be put” (cited in Potts, 2009, p. 106).

Ears thus burn when the term is uttered in pop‐psychological explanation,^6^ or when it characterizes the appeal of tabloid celebrities,^7^ or when self‐help gurus seek to package charisma and sell it to us.^8^ Such handling strips charisma of its social‐theoretical import, reducing its relational dimension to the vanishing point by treating it as a personality trait (Parsons [1963:lxxiii] referred to this approach as “trait atomism”). Moreover, these applications feel excessively casual: the fields of entertainment fandom, romantic exploit, and business gamesmanship are a far cry from the historically consequential political and religious examples that have lent a sense of importance to most academic work on the topic (Collins, 2020; Eatwell, 2014; Smith, 2000).

It is noteworthy that, for all this heat, the sociological polemic against “popular charisma” is most often indirect: it arises as extemporaneous commentary made at the outset of an otherwise academic project. Reinhard Bendix (1967:341), for example, opens his classic essay noting that “The term “charismatic leader” has recently attained widespread and almost debased currency.” Randall Collins (2020:1) begins his book with the observation, “Charisma is a sociological term that went viral.” In this way, charisma's popular valences are deemed necessary to mention, but only as an act of throat clearing that, once performed, allows for quick onward transit to the scholarly task at hand.

This brisk treatment notwithstanding, our knowledge about precisely how “popular charisma” has diverged from sociological operationalization is so far anecdotal, and we know still less about the severity of such departures in quantitative terms. It is therefore difficult to be confident on the basic matter of whether the above objections are warranted or whether academics are being overly protective about their term.

We propose to address “popular charisma” head on. Our aim is to discern and theoretically account for patterns in the relationship between scholarly and vernacular uses of “charisma.” By probing “charisma's” anchorage in common speech, we seek to understand which of its connotative dimensions render it serviceable to wider discourse, and further, whether these dimensions reflect aspects of charismatic power that have not yet been adequately addressed within existing social‐theoretical treatments of charisma. With this approach, we hold in abeyance questions about whether “charisma” is a singular entity that can be defined either correctly or incorrectly. Rather, for our starting point, we recognize that “charisma” is many things to many people, and we seek to explore whether popular instincts about the term's usage—pertaining to its nature, range of applicability, and lines of cultural impact—may in fact hold contributory potential for social theory.

To address these matters, we use machine learning to locate “charisma” within the wider discursive field out of which it arises (and continues to arise) semiotically. We perform this locational procedure within four corpora where “charisma” is salient: Weber's own writings; a collection of post‐Weberian social scientific research; and social media (“X”) collections on “charisma” and its 2023 variant, “rizz.” By ascertaining the meaning structures that discursively suspend “charisma” across multiple dimensions between academic and popular discourse, we provide new insights not just about the cultural and sociolinguistic lifecourse of “charisma” itself, but also about the more general ways that academic and popular discourse interact (Altheide, 2009; Camic et al., 2011).

A series of questions motivate our research. First and most basically, in what ways does “popular charisma” adhere to and/or take leave from “scholarly charisma?” Are these vectors of departure or adherence patterned or predictable, such that we may use them to understand new cultural developments, like the aforementioned “rizz?” Do popular formulations constitute substantive changes to sociological versions, or are they best understood as distinctions without much difference?

Second, do post‐Weber scholarly uses of charisma in fact hew closely to Weber's original formulation, or are they themselves reflective of “popular charisma” as it has developed in the century since Weber's death? In other words, is post‐Weber social science as pure as occasional critics from the “sociology side” would hope it to be?

Third, what might social theorists learn, both from popular figurations of charisma and from the nature of the interaction between its popular and scholastic versions? Weber himself frequently drew on vernacular terms to illustrate the concept,^9^ and while academic and popular meanings have at times diverged from one another, they have doubtlessly informed and influenced one another. We seek to be more intentional about studying and making use of this popular‐discursive “backflow” into sociology.

## 2 METHODS

Generating definitional and dimensional features of “charisma” either in academic discussions or in general discourse requires a systematic analysis of enormous amounts of text which is far beyond the regular human capacity to read and code. Recent developments in machine learning (ML) provide an opportunity to efficiently analyze large volumes of text and discern patterns of semantic meaning that would go unnoticed by human researchers employing traditional methods of coding and thematic analysis. Therefore, we use ML to generate empirical information about different uses of the term, whether in academic or general discourse, which can then be formally compared with one another.

We use Word2vec for this project. Word2vec is the most widely used ML algorithm for the analysis of word embedding. It is a neural network algorithm that can position words based on their surrounding context by inputting large collections of text and outputting a word vector model. In this way, Word2vec is capable of accurately capturing and revealing complex semantic relations by transforming words into vectors (Kozlowski et al., 2019). These vectors can, in turn, be used to identify other words that are to varying degrees synonymous with the term under investigation (in this case, “charisma”). By assessing the nature of synonymity in different discursive contexts, we can then draw inferences about what the term means in these contexts. For example, one can imagine synonyms for the term “eagle,” generated by a Word2vec algorithm trained on birding guidebooks. “Raptor” and “bird” would in this case likely emerge as being variously synonymous with “eagle” in this discursive context, with the former term (raptor) having a higher degree of synonymity than the latter (bird). By contrast, an algorithm trained to produce synonyms for “eagle” within a corpus of golf magazines would likely produce a very different resulting set of words. In this manner, the present method treats our central term (“charisma/charismatic”) as a floating signifier.^10^ Rather than starting with a predetermined conceptualization of “charisma,” we instead use machine learning to discern what the term seems to mean within each respective corpus. We can then compare these meanings across datasets by comparing the lists of synonyms produced for different corpora.

Word2vec has an established track record of ascertaining word analogies and word similarities and has been used in a wide variety of social scientific research. For example, it has been used to calculate associations between different dimensions of “class” over the course of a century (Kozlowski et al., 2019), to analyze the concept of “diversity” in the online institutional presence of elite universities (Rozado, 2019), and to examine cultural shifts along an individualism‐collectivism spectrum in China during the last half‐century (Hamamura et al., 2021). As such, Word2vec, is of great value when seeking to learn about language and its use (Ji et al., 2019).

## 3 DATA SOURCES

To assess the relationship between scholastic and popular‐discursive meanings of charisma, we compared among several corpora, including: (1) Weber's classic texts; (2) social science documents from JSTOR; and X (Twitter) content on (3) “charisma” and (4) “rizz.”

The corpus of Weber's classic texts consists of the following books:

*Economy and Society*
*The Sociology of Religion*
*The Protestant Ethic and the Spirit of Capitalism*
*The Religion of China*
*The Theory of Social and Economic Organization*
*The Agrarian Sociology of Ancient Civilizations*
*The Methodology of the Social Sciences*
*General Economic* *H* *istory*
*The Vocation Lectures* (i.e., “Politics as a Vocation” and “Science as a Vocation.”)

We selected JSTOR as the source for research from social science. We retrieved datasets from Constellate, a platform for text analysis that provides downloading services from JSTOR. 123,531 documents were collected from a variety of social scientific disciplines from 1960 to 2023.

We chose X as the source for popular use of charisma and its contemporary permutation, “rizz” (Orolić, 2023). X is one of the most popular platforms around the world, with wide representation across demographic spectra. The data used in this article is comprised of two subsets which were obtained from two sources respectively. The first subset was gathered by data crawling, through which we obtained 163,030 thousand tweets from 2022 to 2023 which contain “charisma” (*n* = 77,161) or “rizz” (*n* = 85,869). The second sub‐dataset was downloaded from Stanford Network Analysis Project which was established in 2004 to analyze large social and information networks (Leskovec, 2009). This second set contains 476 million tweets from 17 million users, covering about 20%–30% of all public tweets posted from June 1, 2009, to December 31, 2009 (Yang & Leskovec, 2011).

The Twitter‐rizz data proved the most difficult to interpret because much of this content is a product of Internet youth culture's penchant for slang, meme generation, and abbreviation. To assist in the interpretation of the “rizz” data, we therefore conducted a qualitative analysis of a convenience sample of 20 popular press articles that appeared after “rizz” was designated “Word of the Year” by Oxford University Press in 2023. These pieces were especially helpful for deepening and thematizing the results from the quantitative data because they often adopted a translational mode, self‐describing as “explainers” with titles like, “Rizz definition: We explain the Oxford Word of the Year” (*Vox*.*com*), “‘Rizz’ Is Oxford's 2023 Word of the Year: Heer's What it Means” (*People*.*com*), and “What is rizz, and why is it Oxford's word of the year for 2023?” (*NBCNews*.*com*). Without pretentions toward definitiveness (or that “definitiveness” ever obtains with Internet discourse), we are confident that we have grasped the meaning of “rizz” well enough to allow for comparison with other uses of “charisma.”

## 4 RESULTS: COMPARING WORDS CLOSEST TO CHARISMA/RIZZ

To capture the meaning of charisma in different contexts, four Word2vec models were trained using the Weber corpus, the JSTOR corpus, and the Twitter corpora relating to “charisma” and “rizz,” respectively. After such training, we were able to derive words that were to varying degrees synonymous because they appeared in contexts most similar to those which contained “charisma” (or “rizz,” in that case) among the aforementioned corpora. These are presented in Table 1.

**TABLE 1:** Ten words closest to the target word in different data sources.

Data sources	Weber corpus	JSTOR corpus	Twitter corpus	
Target word	“*charisma*”	“*charisma*”	“*charisma*”	“*rizz*”
Top ten words	Routinization	Sohm	Erudition	frfr (“for real”)
	Legitimation	Virtuosity	Confidence	Denji (manga character)
	Depersonalization	Virtuoso	Intellect	Nerdboy (a socially inept boy/man)
	Succession	Inspiration	Genuineness	Powerz (powers)
	Transformation	Follower	Frankness	Skillage (being skilled)
	Kingship	Purveyor	Ambition	Fortnite (video game)
	Heroism	Demagogue	Personality	Wtfness (noun form of “What the fuck”)
	Patriarchalism	Superhuman	Arrogance	Sensay (alt. form of “sensei”)
	Sacramental	Mystagogue	Sophistication	Grimace (purple McDonalds character)
	Attenuate	Weberian	Professionalism	Phag (“fag,” but less pejorative)

The table shows clear semantic divergences among the four corpora. We interpret each set of results below, in turn.

### 4.1 The Weber corpus

For the Weber corpus, one can see that there is a preponderance of terms that reflect Weber's overriding preoccupation with “routinization”—the stabilization of charisma within social structure. If the life course of charisma can be divided roughly into two phases—its explosive inceptive moment, followed by its end through routinization—Weber was drawn most consistently toward theorizing the latter stage, which he regarded as an inevitability due to charisma's self‐limiting nature:

Every charisma is on the road from a turbulently emotional life that knows no economic rationality to a slow death by suffocation under the weight of material interests: every hour of its existence brings it nearer to this end

(Weber, 1922(1978), p. 1120).

The sections in *Economy and Society* describing charisma in its own terms are relatively short when compared with the lengthy chapter, “Charisma and its Transformation” (1922(1978), pp. 1121–1148) which describes a variety of ways in which charisma either traditionalizes or rationalizes. Listed terms like “depersonalization,” “succession,” “transformation,” “patriarchalism,” “attenuate” and of course “routinization” thus reflect Weber's preoccupation with charisma's imbrication within structure.

To some degree, this emphasis also reflects aspirations Weber harbored for his larger project of relating charisma, as one form of authority (*herrschaft*), to the rational‐legal and traditional forms. This is the tripartite theoretic frame for “legitimation” (term two on the list) that dominates *Economy and Society* and that serves as a theoretical back‐structure for his work on the religions of India, China, and Israel.

We can also note a couple of themes that are conspicuous in their absence from the Weber list. For example, Weber's departure from the original Christian conceptualization of charisma is evident in that only one term—“sacramental”—seems to derive from the term's original Greco‐Christian context. And no word among the top 10 (or even the top 20) is relevant to what he sometimes described as “religious” or “magical” charisma. This too is indicative of Weber's intentions—in this case, to broaden charisma into an analytic category that would be relevant beyond the realm of religion:

[S]ince [church historian Rudolph Sohm] developed this category [charismatic authority] with regard to one historically important case—the rise of ecclesiastic authority of the early Christian church—his treatment was bound to be one‐sided from the viewpoint of historical diversity

(1922[1078]:1112).

Weber's approach is thus programmatically general. The absence of terms that could be exclusively identified with any particular case speaks to his choice to populate his descriptions of charisma with many disparate examples, with none playing a leading or dominant role in the representation of charisma. This reflects Weber's concern with clarifying concepts in such a way that they can be used effectively in socio‐historical analysis across many substantive areas.

Also absent from the list are “personality qualities” as such. When Weber does describe charisma in its personalized form, he tends to do so through generalized roles (“kingship,” for example), rather than with words for specific character traits—the latter being more common in both the JSTOR and social media corpora (see below).

In three ways, then, a clear sense of the nature of Weber's charisma—which will be readily recognizable to Weber scholars—emerges: (1) Charisma is something that is disruptive of structure but short‐lived and fated toward transformation into structure. (2) Weber's charisma is not principally associated with the originating case of Christianity or with specifically religious or magical contexts. Instead, it is expressly general. Finally, (3) Weber's charisma is not described as a personality trait. When Weber does describe charisma in a personalized form, he is likely to refer to status roles that reflect a generalized structural location (i.e., “kingship”) or states that imply attributional processes of valorization by follower‐admirers (i.e., “heroism”).

### 4.2 The JSTOR corpus

Unlike with the Weber corpus, the JSTOR corpus gives considerable prominence to terms which directly reference the qualities of leaders. Terms like “virtuoso/virtuosity” and “superhuman” suggest a focus on the skills and/or attributes of someone who possesses charismatic power. While Weber himself used both of these terms at times (i.e., 1922(1978):241, 649), their relative prominence in the JSTOR corpus suggests a shift away from Weber's socio‐structural focus and toward what is sometimes criticized as “leader‐centrism” in charisma research (Joosse, 2017; Reed, 2013; Willner, 1985).

Like with Weber, vocabulary associated with role statuses is present. But in the JSTOR corpus, it is much more prominent. “Follower,” “inspiration,” “demagogue,” “purveyor,” and “mystagogue” all denote relational positions within the phenomenon of charismatic interaction, either on the follower‐side (in the first two terms) or in the leader role (the latter three). These terms reflect a preoccupation among post‐Weberian social scientists with the “charismatic bond”—a phrase Weber himself never used, but which dominates social‐psychological and management research literatures and which features prominently in several subfields pertaining to new religious movements (i.e., Jacobs, 1987), social movements (i.e., Madsen & Snow, 1991), political extremism (i.e., Eatwell, 2014; Gendron, 2017), and populism (i.e., Albertazzi & McDonnell, 2008).

One side issue worth noting for the JSTOR corpus is the appearance of [Rudolph] Sohm at the top of the list. This means that the Lutheran Jurist and church historian almost never appears in contexts where “charisma” does not also appear. Clearly, this owes to Weber's own influence on the JSTOR writers, since Weber (1922(1978):216, 1112) twice credits Sohm's work as an inspiration. Thus, while Sohm is firmly established in the story of charisma's origins, he persists in social science *only* in this manner. Peter Haley (1980:1) remarked upon the irony that, while the 1934 edition of the *Encyclopedia of Social Sciences* contained a biographical entry on Sohm but no mention of ”charisma,” the 1968 edition of the same encyclopedia excluded the entry on Sohm while including Edward Shils' five‐page article on “charisma.” In this way, Sohm, once widely recognized and respected as a historian in his own right, now persists merely as a discursive ride‐along, hitched to the runaway success of Weber's “charisma.”

### 4.3 The Twitter‐Charisma corpus

The Twitter‐Charisma corpus is striking in that, without exception, the terms all relate to personality traits or attributes. Standalone individuals are the most natural referent for terms like “erudition,” “confidence,” “intellect,” “frankness,” “ambition,” “arrogance,” and “sophistication.” This suggests a more individualized conception of charisma: those who are using “charisma” this way are either less cognizant of, or simply less concerned with the relational and sociostructural dimensions that are the focus of sociological explorations.

A closer examination reveals that the terms cohere around three distinct connotative domains. The first relates to traits suggesting personal excellence. “Erudition,” “intellect,” “sophistication,” and “professionalism” all stand out in this regard. The second domain involves qualities that approximate various elements of assertiveness. “Confidence,” “ambition,” and “arrogance” fall into this category. Finally, there are terms that relate to authenticity or transparency, broadly conceived. “Genuineness” and “frankness” represent this aspect.

Charisma according to Twitter thus seems to manifest as a relatively simple character sketch: the charismatic individual is intelligent and well‐spoken. They are self‐assured to the point of overconfidence or arrogance. Finally, they are relatively genuine or transparent in the way they present themselves. Moreover, within the Twitter‐Charisma corpus, the predominant range of action pertains to personal achievement—charismatic individuals are an incarnation of self‐help mantras about individual improvement, positive self‐regard, and character strength. Elements that were so central to Weber's version of the concept, like its macro‐structural efficacy, its penchant for moral transformation, and its revolutionary potency, are nowhere to be found in Twitter‐Charisma's discursive field.

### 4.4 The Twitter‐Rizz corpus

Because it is a new phenomenon, it is important to provide some background on “rizz.” ”Rizz” first emerged among New York influencers like Kai Cenat, Duke Dennis, and Silky in 2021, picking up popularity in online subcultures through 2022 prior to its viral eruption in 2023. From its earliest iterations through to the present, “rizz” served as a descriptor for romantic skill—approximately synonymous with “game” or “chatting someone up.” In the first half of 2021, for example, Twitch streamers Cenat and Silky began posting streams in which they would silently observe each other's e‐dates, assessing and critiquing each other's “rizz” (Carry, 2023). This format—involving romantic pursuit, observation, and critique—constitutes a presentational structure that consistently characterizes the appearance of “rizz” in Internet culture.

The prevalence of this format allows for two observations. First, part of the traction of “rizz” clearly owes to voyeuristic appeal. People find it enthralling to peer into and assess others' romantic moves, and this “look‐see” aspect drives viewership on content sharing platforms. Thus, while titles for videos on the topic often adopt a promissory mode—touting the ability to show viewers “how they can have rizz” (Oxford, 2023)—in actuality, this is not instructional material. Instead, people are watching most often for no other reason than that it is fun to partake in the drama besetting those who go out on a romantic limb, hoping for acceptance while risking rejection.

Second, it is evident from this format that rizz is most captivatingly comedic when it *fails*. When the pickup lines don't land; when the approaches are awkward or obviously contrived; or when performances fall flat and are met with eyerolls and laughter—this is what generates views and commentary. In this sense, we can say that “bad rizz” makes for good rizz content.

We would be mistaken to interpret this vicarious enjoyment at others' failure as being wholly mean‐spirited, however. Rather, a camaraderie is frequently found in these spaces in which creators and commenters commiserate with one another over the fact that they too “lack rizz” or have “l‐rizz” (loser rizz). Words from the Twitter‐Rizz list seem to reflect this “loser‐solidarity.” “Nerdboy” suggests someone who would not do well romantically. “Grimace” is a purple glob‐shaped McDonalds mascot who is jokingly described in memes as having good pickup lines. “Denji” is a manga character known for intense infatuation with women (Makima and Reze) who never reciprocate his attention, treating him like a pet or a tool. Finally, “Phag” (a less harsh version of the homophobic slur) is suggestive of someone who, while perhaps not actually being gay, would nevertheless struggle to find success with women.^11^ Like Peter Parker prior to his transformation into Spiderman (see below), non‐charismatic or pre‐charismatic states are often characterized using diminutive descriptors: colloquialisms like ‘runts’, ‘half‐pints’, ‘small fries’, or ‘sonnies’ frequently serve to signal the status of individuals prior to their charismatic/heroic realization (Joosse & Zelinsky, 2022, p. 1077).

Thus, while on its face the Word2vec list seems somewhat paradoxical in that it is populated by words denoting a distinct *lack* of romantic charm, this valence of negation can be explained if we recognize that, within Internet subcultures, “rizz” tends to be an object of fascination precisely for those who feel they lack it. This aspect—wherein rizz is discussed primarily in the context of its absence or in relation to its failure—thus resembles wider trends in internet‐mediated masculinity, where a lack of romantic success characterizes concepts like “beta males” and “incels” (Ging, 2019). These features also distinguish “rizz” from the “charisma” of the Twitter‐Charisma corpus, insofar as the latter is much more straightforwardly positive about its central object.

“Rizz lack” was in fact instrumental to the term's 2023 viral explosion. In June of that year, Tom Holland, the actor best known for playing Spider‐Man in several iterations of the Marvel film franchise, gave an interview with *BuzzFeed* in which he discussed “rizz” in the context of his romantic relationship with Zendaya, one of Hollywood's most prominent young actresses. In the interview, Holland smiled as he confessed,

I have no rizz whatsoever; I have limited rizz…. I need you to fall in love with me *really* for it to work. So [I play the] ‘long game’—probably [by] making a movie with each other [*sic*]. It definitely helps when the characters you’re playing are falling in love with one another—you can sort of blur the lines [between film roles and reality] a little bit. That’s kind of where my rizz is at, and you know I’m locked up [in a relationship] so I’m happy and in love. So, I’ve got no need for rizz.

Holland's interview sparked intense discussion and occasioned a flurry of memes. Oxford's data showed a fifteenfold increase in the term's usage in the month of Holland's interview, and his comments were referenced by many mainstream outlets as leading to the term's viral uptake.^12^

“Rizz lack” is very much on display in Holland's quote. Rather than professing to have rizz, Holland instead cites circumstantial factors (playing love interests in three *Spider‐Man* films) as being decisive for “locking him up.” Moreover, rather than crediting his rizz‐based “skillage” or “powerz” (words 4 and 5), he instead cites a much more traditional, timeworn basis for his and Zendaya's attraction—real love.

In the wake of #MeToo's critical appraisal of power dynamics in Hollywood, one could imagine pointing out that there is something unseemly about “blurring the lines” with a co‐worker in the manner Holland describes. But takes of this sort were largely absent in discussion surrounding the interview, and instead Holland was widely (and jokingly) praised as being “cute” for this admission that he lacked rizz. Moreover, his love for Zendaya was consistently commended as being “true” and “adorable” because it was not the product of rizz‐style gamesmanship. The affection for Holland was in this sense not dissimilar from that enjoyed by his film‐based alter‐ego Peter Parker (to blur the lines still further). Both cases present us with the nice‐guy/loser^13^ whom we are happy to cheer on as he strives for greatness—and to get the girl.

## 5 COMPARING VECTORS OF “CHARISMA”

**FIGURE 2:**
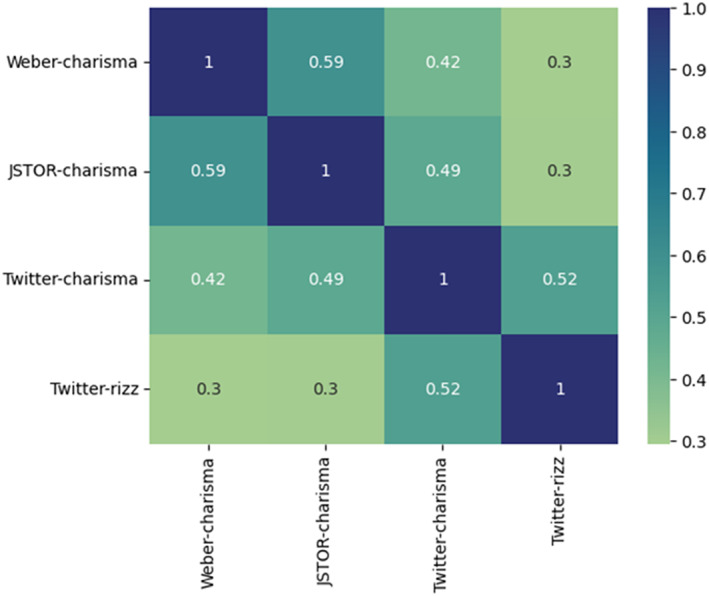
Cosine similarities of “charisma” among the four corpora.

The above analysis used machine learning to quantitatively derive terms that were then used for descriptive elaboration and qualitative comparison among the corpora. To sharpen the quantitative basis for such comparisons, we utilized Procrustes matrix alignment (Hamilton et al., 2018), which allowed us to align vectors of “charisma” and “rizz” from the different corpora along the same coordinate axes. Drawing on the two quantitative laws of semantic change identified by Hamilton et al. (2018)—namely the *law of conformity* and the *law of innovation*—we align the vectors for the various terms with one another, such that we can compare the relative rate of change and conformity among these terms, as they present in the different corpora. This procedure allows us to say that the higher the cosine similarity is between any two corpora, the closer the usage of “charisma” or “rizz” is between those corpora. Figure 2 presents these comparative results.

One can see that the cosine similarity between the Weber and JSTOR corpora is 0.59; between the Weber and Twitter‐Charisma it is 0.42; and between Weber and Twitter‐Rizz the cosine similarity is 0.3.

These results indicate that, among the three corpora, the meaning structure of the JSTOR corpus resembles Weber's charisma most closely. However, the margin through which JSTOR wins this contest over Twitter‐Charisma is not very large (0.59 to 0.42). Further, when one considers Twitter‐Charisma's semantic similarities with the various corpora, we can see that the differences between it and the two scholarly corpora (Weber and JSTOR) are negligible (0.42 vs. 0.49). This means that Twitter‐Charisma, as a cluster of semantic meaning, is close to being interchangeable, in terms of degrees of synonymity, with Weber's and JSTOR's charisma.

When it comes to Twitter‐Rizz, the discrepancies are starker. Twitter‐Rizz departs in equal measure from both Weber and JSTOR charisma (0.3 and 0.3), while being more similar with its Twitter‐Charisma counterpart (0.52). This allows us to conclude that, although rizz derives from charisma etymologically, and even though this derivation is recent, it has deviated the most from academic uses. As such, this finding conforms with both our qualitative and quantitative analyses above. As President of Oxford Languages Casper Grathwohl explained in a press release:

Rizz is a term that has boomed on social media and speaks to how language that enjoys intense popularity and currency within particular social communities…can bleed into the mainstream. This is a story as old as language itself, but stories of linguistic evolution and expansion that used to take years can now take weeks or months. The spike in usage data for rizz goes to prove that words and phrases that evolve from internet culture are increasingly becoming part of day‐to‐day vernacular…

Through such processes, rizz is thus quickly departing from “charisma,” becoming a discursive creature unto itself.

## 6 DISCUSSION

When sociologists were polled at the end of the 20^th^ century about work that most influenced them, they overwhelmingly selected *Economy and Society* (ISA, 1997), a book where Weber is preoccupied with theorizing authority (Roth, 1968, p. xciii), and where he devotes many pages to defining and describing charismatic power. Given this cherished status, one can understand how it might feel like a bit of a profanation when “charisma”—a sacred object if sociology ever produced one—is handled without due care.^14^

Our findings suggest, however, that rather than hewing closely to the Weberian formulation, scholars since Weber have themselves participated in what they sometimes otherwise decry as charisma's vulgarization, steering it in some of the same directions that characterize contemporary trends in popular use. Such tendencies have been discussed in previous reviews of charisma research (Joosse, 2014; Smith, 2013), but the distinguishing advantage of the present approach is that it allows for a systematic description of sematic departures among various versions of the term, as well as quantitative comparison of the relative severity of such departures.

Without this feature, it would be possible to create any number of narratives about the sociological propriety of various actors' uses of “charisma.” For example, Weber himself has frequently been criticized for advancing a leader‐centric, even Carlylian, vision—a charisma that is putatively a product of singular individuals (Bourdieu, 1987, pp. 129–131; Cavalli, 1987, p. 318; Gerth & Mills, 1946(1958): 53). Based on such characterizations, these critics often position their own interventions as helping to “sociologize” Weber himself (Bourdieu, 1987). Our algorithmic examination of a large corpus of his work confirms that, contrary to the assertions of such authors, Weber was unmistakably sociological; preoccupied with the relational and structural conditions that give rise to charisma, and more than this, the social forces that bring charisma to its end.

### 6.1 Three spectra of distinction

The most notable takeaway from our Word2vec analyses is that JSTOR's social scientific uses exist in an intermediate position vis‐à‐vis Weber's version and the popular/social media versions. This intermediate position manifests along three distinct spectra. We will describe each of these in turn.

#### 6.1.1 Structural versus individual ranges of efficacy

The first spectrum pertains to the existence, or lack thereof, of a focus on charisma's *structural efficacy*. On the Weberian side, we find a charisma that is dialectically related to social structure: in its revolutionary mode, charisma is destructive of structure, but in the longer term, it is fated toward imbrication within social structure. The narrative arc for charisma at this pole is thus driven by charismatic protagonists and their structural agonisms.

This contrasts starkly with what we find at the other end of the spectrum, where charisma manifests nearly exclusively as an individually‐agentic phenomenon. Here, charisma is primarily conceived as a factor that makes personal advancement more or less likely, whether this be in the realms of business, love, or some other personal domain. Users of “charisma” at this end of the spectrum thus clearly have different purposes from those on the Weberian side.

As described, the JSTOR corpus reflects a position between these poles. Rather than words that directly address structural efficacy, JSTOR terms relate to role‐statuses that only obliquely imply a larger structural field of play. At other times, the terms are quite like those from the social media lists in that they convey a comparatively essentialist focus on leader qualities.

#### 6.1.2 Morally neutral and transformative versus morally positive and conservative

The second spectrum relates to the role of normativity in descriptions of charismatic power. On one end (again, the Weberian end) we find an approach that is intentionally agnostic about the ultimate value of charisma's sociocultural impact—charisma is neither “good nor bad.” For this reason, classic perspectives can easily describe both Hitler and Martin Luther King Jr. as “charismatic,” and this ability derives from Weber's aim of producing concepts that are “value‐free” (1922(1978):1113). These approaches also emphasize that charisma is morally transformative: it denatures and revolutionizes the moral‐cultural systems out of which it arises. Classic/Weberian charisma is often characterized as being “antinomian” for this reason.

On the other end are approaches exhibiting a largely positive appraisal of charisma arising from the presumption that it will increase individuals' ability to be successful in their pursuits.^15^ This positive estimation is complicated somewhat in that while Twitter‐Charisma is straightforwardly positive, Twitter‐Rizz tends to be mediated by a posture of removal, as described above. This complexity notwithstanding, the overall estimation of charisma by social media users is positive.

Once again, the JSTOR corpus presents an evaluation that appears on a middle ground between these two poles. That is, while there is a pretense of analytical removal in much of this work, the terms produced, which often relate to desirable individual qualities, reveal a positive estimation of what charisma is and does. No doubt this partly stems from the motivations behind such research. Business management literature, for example, tends to study charisma with the aim of producing more effective management policy.

#### 6.1.3 Relational‐interactive versus individual‐traitist charisma

The third spectrum spans the divide between viewing charisma as having either a relational or individualist ontology. The relational side stresses that charisma is socially produced, whether at the macro‐level of “charisma hungry” populations (Friedland, 1964), or at the micro‐range involving “charismatic counter‐roles” (Joosse, 2018). The most personalized valences of this relational charisma tend to be described in terms of role‐statuses which themselves imply broader sociological processes of distinction. In this vision, leader “qualities” as such are seldom mentioned.

At the other end we have what Parsons' (1963:lxxiii) described as “trait atomism.” Here, charismatic leaders can be captured by way of a character sketch comprising an assemblage of personal qualities. In this vision, charisma is either something one acquires through personal cultivation, or it is simply an inherent endowment.

Here too, we find that the JSTOR set occupies an intermediate position. While the corpus makes frequent references to role statuses, it combines these with a considerable prominence given to terms that directly reference the qualities of leaders.

**FIGURE 3:**
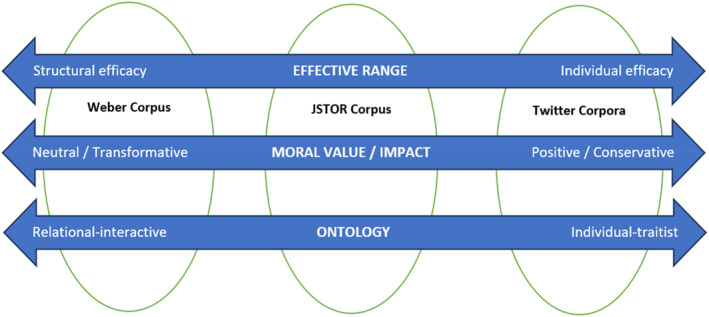
Three distinguishing dimensions: charisma's (1) effective range, (2) moral value and impact, and (3) ontology.

These three spectra are visualized in Figure 3, below.

So, what to do with this information? On the one hand, these results could be used to endorse those who want to protect their term by erecting strong boundaries between what they regards as ”true” charisma (in the sociological sense) and what otherwise might be described as “mere celebrity,” “idolatry,” or “pseudocharisma” (see Smith, 2013, pp. 26–32 for a discussion of this trend).^16^ As researchers who find the classic statements almost endlessly edifying, we have some sympathy for this impulse. But ultimately, we believe this path is unwise, for two reasons.

The first has to do with lost opportunity. In the century since Weber lived, sociology's remit has expanded to illuminate a host of social dimensions—emotions, culture, the body, media, gender, among others—the analysis of which could only have been envisioned in the vaguest of terms from Weber's position on the discipline's ground floor. A protectionist approach thus risks freezing charisma scholarship within an anachronistic disciplinary paradigm, foreclosing the possibility for the concept to travel alongside and interact with important developments in the field. Thus, while we agree with sociology's partisans that Weber's formulation stands unmatched as the most well‐realized vision of charisma yet produced, we believe that its power as theory *qua* theory lies precisely in its ability to transcend the historical circumstances through which it was originally extruded. To reserve the application of charisma for phenomena that would be considered “serious” in a traditional sense therefore risks consigning the concept to ever greater irrelevancy within sociology, even as its popularity grows without.

Instead, we would prefer to reverse the focus, entertaining the possibility that “popular charisma” might be delivering useful signals about the changing nature of contemporary social life. Stephen Turner (2003, p. 6) observed that “assessing the contemporary relevance of charisma…. is not a matter of simply looking at the reception‐history of Weber's use of the term, or its application to empirical sociological research. [Rather,] one must understand the background tectonic shift in the conditions of social and political life” that has contributed to this relevance. Emotions, celebrity, and embodiment certainly seem important to contemporary concerns about charisma's relevance, whether this be with polarization (Davis & Vila‐Henninger, 2021), populism (Joosse & Zelinsky, 2022; Street, 2019), or affective leadership in social media (Joosse & Zelinsky, 2023; Reh et al., 2017).

The second reason to resist protectionism of the above sort is because we feel it is simply unnecessary, so long as we observe a classic anthropological distinction between emic and etic viewpoints on social research. Here, Weber's (1922(1978):241) original definition is helpful:

The term ‘charisma’ will be applied to a certain quality of individual personality by virtue of which he *is considered* extraordinary and *treated as* endowed with supernatural, superhuman, or at least specifically exceptional powers or qualities. These are such as are not accessible to the ordinary person, but *are regarded as* of divine origin or as exemplary, and on the basis of them the individual concerned *is treated* as a ‘leader.’

Emphases have been added to the quote above to prevent Weber's passive voice from obscuring the sociological point he was clearly making. That is, in every line Weber fully anticipates that for nearly all involved charisma will appear to be—and will be spoken about as being—an individual's essential quality. To begrudge popular users for this mode of apperception, taking issue with their lack of analytical distance in such situations, would thus be somewhat odd, since Weber's theory of charismatic formation is predicated on just such “uncritical” appraisals of charismatic endowment. With this emic/etic distinction in hand, we can therefore resist accepting unreconstructed essentialisms and individualisms as they apply to charisma, but also understand that those who are using the term in these ways are using it as Weber recognized it would be used, however unexciting this fact may be to the public.^17^

To illustrate this last point, the first author will forgo the third person to recount a conversation, with a former ASA President, that speaks to the need for clarity on this matter. The topic was (as it so often is) Donald Trump, and what theories might help to clarify the Trump phenomenon. When I mentioned charismatic authority, my interlocutor replied that, although she agreed with the interpretation in a formal sense, she also had misgivings. Then, she asked with evident incredulity, “But do *you really* find him charismatic?” This turn in the conversation—especially the way she leaned into “*you*” and “*really*” here—startled me. I realized that I was now being asked something different and more personal; something that carried with it a hint of accusation. “Yes…,” I answered, but I realized quickly that I should qualify my response: “But this isn't my personal evaluation.” Sensing the need for further exculpation, I appealed to authority: “Others, like Arlie Hochschild and Steven Lukes have interpreted him this way as well.” The better answer—the one I regret not giving—would have involved defending the Weberian tradition of observing a distinction between emic and etic viewpoints on such matters. With this distinction, we will be able to better preserve the sociological utility, if not the virality, of this most classic of classic concepts.

## Data Availability

The data that support the findings of this study are available from the corresponding author upon reasonable request.

## ENDNOTES

^1^
“Charisma” appears 17 times in the New Testament. All but one of these instances (1 Peter 4:10) occur in Paul's letters (Conzelmann & Zimmerli, 1974, p. 403).

^2^
Weber (1922) cites Karl Holl and Rudolph Sohm as theological forebearers whose uses of charisma were important to the development of his sociological vision (p 216; 212). Theologian Friedrich Schleiermacher (1768–1834) was another important theologian who used the term prior to Weber and who was read by Weber as a student (Potts, 2009, p. 109). For the connection between “Caesarism/Caesaropapism” and “charisma” in 19th century political debate, see Baehr (2008).

^3^
Although Weber briefly mentions charisma at the end of *The Protestant Ethic and The Spirit of Capitalism* (1905/1920), his main work on the topic began with articles he produced for the *Archiv für Sozialwissenschaft und Sozialpolitik* which comprise his “world religions” studies, conducted from 1915 to 1919 (1915(1951), 1916(1958), 1919(1952)), his *Politics as a Vocation* 1919(1946) lecture, and the posthumous *Economy and Society* (1922).

^4^
Parsons went somewhat further, crediting Weber with coinage (1920(1958):281, n.105; 1937(1968):564, n. 5).

^5^
This may be overstating things somewhat. Talcott Parsons' (1937(1968)) early efforts to introduce Weber to American sociology were clearly important also. Be that as it may, the role and influence of “popular charisma” on its academic cousin remains an important topic of investigation.

^6^
Pogosyan (2019).

^7^

*New York Times* columnist Russell Baker noted that amid its popular uptake charisma was “debased to mean something much closer to what used to be called, back in the days of the big Hollywood studios, ‘star quality’” (quoted in Derman, 2012, p. 214).

^8^
See the Charisma Training Academy (https://www.charismatrainingacademy.co.uk/), The Art of Charm (http://theartofcharm.com/), and Charisma on Command (https://www.youtube.com/@Charismaoncommand).

^9^
In addition to the theological term “charisma,” Weber also used “maga” (from ancient Persian), “orenda” (from the Iroquois), “mana” (from Polynesian civilizations), and “berserk” (from Norse religion) to describe charisma (1922(1964):400; 1919(1952):411).

^10^
We use the term in the sense meant by Lévi‐Strauss (1987, p. 55); namely, words that “like algebraic symbols, occur to represent an indeterminate value of signification, in itself devoid of meaning and thus susceptible of receiving any meaning at all.” interestingly, Lévi‐Strauss (1987, p. 63) himself describes *mana* (from Polynesian) and *orenda* (from Iroquois) as floating signifiers. These two words, in turn, were used by Weber as synonyms for charisma (see note 9, above).

^11^
In our observations, rizz nearly always manifests in heteronormative contexts as a function of masculine ability, referring to the capacity of men to attract women (Wright, 2023). For more on gender and charisma, see Joosse and Willey (2020).

^12^
These included *The New York Times*, *Time*, *People*, *The Atlantic*, *The Guardian*, *Vox*, *NBC*, *CNN*, *CBS*, and *BBC*.

^13^
Spider‐Man was introduced in the August 1962 issue of *Amazing Fantasy* with the following dialog: “Though the world may mock Peter Parker the timid teenager, it will soon marvel at the awesome might of Spider‐Man!”

^14^
Phrases like “the Marx‐Durkheim‐Weber trinity” (Murray, 1996, p. 13) are in this sense only partly rhetorical because they reflect the real capacity for certain figures and concepts to glimmer with totemic value within the field, speaking to a reverence for founders that cannot be found in, say, psychology (Coser, 1981, pp. 181–182).

^15^
It may be surprising to some to see that, in these findings from Twitter, there is very little representation of what might be referred to as “negative” aspects of charisma, as exhibited by certain figures like leaders of cults which have committed mass suicide (Marshall Applewhite and Jim Jones, for example) or dictators who, while seemingly widely acknowledged as being charismatic, are also personally responsible for huge death tolls (Hitler and Mao, for example).

^16^
It is interesting to note that Weber himself frequently sought to make distinctions between what he called “pure” charisma and its lesser forms (1922[1978], pp. 216, 219, 244, 263, 1113–1114). These efforts on his part relate, however, to his concern with the degree to which particular historical examples exemplify the ideal type. Weber (1922 [1978], p. 295) was unconcerned with whether putatively charismatic qualities were “actual, alleged, or presumed,” preferring instead to focus on how social processes of attribution deliver charismatic authority to particular leaders. Whether certain qualities, whether physical or psychological are more or less amenable to charismatic attribution is an interesting question that remains open.

^17^
Sociologists, in this sense, are prone to serving as something of a spoilsport with their analyses. See, for example, Palmer (2001, p. 107): “When I told the sannyasis at my neighborhood Rajneesh Meditation Center in 1985 that I wished to do a sociological study of their commune, they rolled their eyes. “Talk about missing the point!” one remarked.”
